# Cognitive Intervention Strategies Directed to Speech and Language Deficits in Primary Progressive Aphasia: Practice-Based Evidence from 18 Cases

**DOI:** 10.3390/brainsci11101268

**Published:** 2021-09-25

**Authors:** Thais Helena Machado, Maria Teresa Carthery-Goulart, Aline Carvalho Campanha, Paulo Caramelli

**Affiliations:** 1 Programa de Pós-Graduação em Ciências Aplicadas à Saúde do Adulto, Faculdade de Medicina, Universidade Federal de Minas Gerais, Belo Horizonte 30130-100, MG, Brazil; caramelli@ufmg.br; 2 Grupo de Pesquisa em Neurologia Cognitiva e do Comportamento, Departamento de Clínica Médica, Faculdade de Medicina, Universidade Federal de Minas Gerais, Belo Horizonte 30130-100, MG, Brazil; aline.campanha@gmail.com; 3 Av Prudente de Morais, 290-Sala 1106, Belo Horizonte 30380-002, MG, Brazil; 4 Grupo de Estudos em Neurociência da Linguagem e Cognição, Núcleo Interdisciplinar de Neurociência Aplicada, Centro de Matemática, Computação e Cognição da Universidade Federal do ABC, São Bernardo do Campo 09210-580, SP, Brazil; teresa.carthery@gmail.com; 5 Grupo de Neurologia Cognitiva e do Comportamento, Divisão de Clínica Neurológica, Hospital das Clínicas da Faculdade de Medicina da Universidade de São Paulo, São Paulo 05403-000, SP, Brazil; 6 INCT-ECCE (Instituto Nacional de Ciência e Tecnologia sobre Comportamento, Cognição e Ensino), Rodovia Washington Luís, Km 235, São Carlos 13565-905, SP, Brazil

**Keywords:** primary progressive aphasia, treatment, speech and language therapy, intervention, cognitive rehabilitation

## Abstract

Background: Practice-based evidence can inform and support clinical decision making. Case-report series about the implementation of programs in real-world clinical settings may contribute to verifying the effectiveness of interventions for treating PPA in specific contexts, as well as illustrating challenges that need to be overcome. Objective: To describe and provide practice-based evidence on the effectiveness of four cognitive rehabilitation programs designed for individuals with PPA and directed to speech and language impairments, which were implemented in a specialized outpatient clinic. Methods: Multiple single-case study. Eighteen individuals with different subtypes of PPA were each assigned to one out of four training programs based on comprehensive speech and language assessments. The treatments targeted naming deficits, sentence production, speech apraxia, and phonological deficits. Pre- and post-treatment assessments were undertaken to compare trained and untrained items. Gains were generalized to a different task in the first two types of intervention (naming and sentence production). A follow-up assessment was conducted 1–8 months after treatment among 7 participants. Results: All individuals presented better performance in the trained items at the post-test for each rehabilitation program accomplished, demonstrating that learning of the trained strategies was achieved during the active phase of treatment. For 13 individuals, statistical significance was reached; while for five, the results were maintained. Results about untrained items, generalization to other tasks, and follow-up assessments are presented. Conclusions: The positive results found in our sample bring some practice-based evidence for the benefits of speech and language treatment strategies for clinical management of individuals with PPA.

## 1. Introduction

Primary progressive aphasia (PPA) is characterized by gradual deterioration of language with relative preservation of other cognitive functions and functional independence, except for situations in which language is critical [1,2].

The international consensus for diagnosing PPA [2] defined three clinical variants (agrammatic/nonfluent, semantic, and logopenic). Around 20 to 35% of individuals with PPA do not fit into these three main variants and are named non-classified or mixed PPA (mxPPA) cases [3,4].

Symptom onset may occur before the age of 65, with a devastating effect on functionality. In the absence of effective pharmacological treatments [5], there is increasing interest in other approaches, particularly behavioral interventions, focusing on communication aspects or specific speech/language deficits. Relative preservation of other cognitive functions, including episodic memory [2,6], enables implementation of SLT, given that individuals with PPA are usually aware of their difficulties and can engage more independently in the activities proposed, with lower demand for support from caregivers, compared with subjects with predominantly episodic memory impairment, who have greater difficulty in learning new content.

Non-pharmacological interventions in PPA can be classified into those directed to the deficits (e.g., anomia, agrammatism, phonological working memory or speech apraxia) and functional interventions (environmental modifications and compensatory strategies). Positive results were reported in most studies but, to be recommended, treatments require further investigation regarding their effectiveness [7]. Most evidence derives from case studies or series [7,8] and randomized-controlled studies with larger samples are needed in order to increase the level of evidence, as there is no consensus regarding types and duration of interventions. However, compared with dementia syndromes (e.g., Alzheimer’s disease), PPA is a rare condition with heterogeneous clinical profiles. Implementation of randomized controlled studies requires a multicenter effort to unify assessment and treatment protocols in order to gather evidence from larger samples. On the other hand, SLT practice requires individualized intervention plans that are adjusted to the context, resources and individuals’ and families’ preferences. Reporting the results from treatments that were implemented can also contribute to the level of practice-based evidence. Practice-based evidence can inform and support clinical decision making and is obtained from several sources, including case reports or case series in “real-life” clinical settings [9].

### 1.1. Research-Based Evidence on Non-Pharmacological Treatments for PPA

#### 1.1.1. Interventions Directed to Lexical Retrieval/Semantic Deficits

Lexical retrieval and/or semantic deficits are common features of PPA syndromes and may predominate over other language or cognitive impairments for long periods. Subsequently, communication becomes markedly affected by word production and comprehension deficits, difficulties in sentence production (agrammatism or paragrammatism) and/or in syntactic comprehension. Speech may also be affected by apraxia of speech and dysarthria [1].

Lexical retrieval treatment is the most widely applied approach [7,10], independently of the clinical variant, given that individuals with PPA usually manifest anomia or word misuse (i.e., lexical retrieval and semantic deficits) with greater or lesser severity. The goal of this treatment is to restore and maintain retrieval of core vocabulary items for as long as possible.

Subjects with PPA are able to relearn target vocabulary during the active phase of treatment and to maintain gains for varying periods after the intervention [11,12,13,14,15,16,17]. Learning may be generalized to untrained stimuli [13,15,16,18,19,20]; however, these findings are still inconsistent. Rising [21] and Croot [8] reported immediate treatment gains in most individuals, and maintenance of gains (months to years) in some individuals with ongoing treatment.

Beales et al. [22] showed that relearning was the most prominent mechanism of change in PPA, followed by stimulation. Reorganization and cognitive relay were less observed. Given the progressive nature of PPA and the urgency of maintaining the preserved vocabulary, perhaps only items that are relevant to daily life should be included in treatment sets [10,18].

Meyer et al. [23] used the term prophylaxis for stimuli that were consistently named correctly (prophylaxis items) and the term remediation for those that were consistently named incorrectly (remediation items) at baseline. Studies on treatment for anomia in PPA have typically focused on remediation of words that could not be named at baseline, rather than on prophylaxis of words that could be named. However, prophylactic treatment may also have positive effects. Reilly [24] defended maintenance of known words over reacquisition of forgotten knowledge regarding semantic treatment paradigms. Studies investigating maintenance of treatment gains have suggested that retrieval accuracy can be maintained (prophylaxis items) or improved (remediation items) with long-term treatment (six months or more) [17,23,24,25,26,27].

Volkmer et al. [28] explained that PPA requires a “staging approach”, in which “impairment-based interventions” (focusing on remediation and rehabilitation) should be implemented at early stages, while compensatory strategies (with the goal of developing strategies to facilitate completion of a particular task) should be implemented after restoration has failed and language skills are lost.

#### 1.1.2. Interventions Targeting Speech and Sentence Production

These interventions are offered to nonfluent subjects and are aimed at syntax training with different approaches, as shown below. Schneider et al. [29] examined the effectiveness of verbal plus gestural treatment on acquisition and generalization of verb tenses in sentence production in one individual and showed that improvement in the production of sentences was achieved through using trained verb tenses.

Andrade-Calderón et al. [30] analyzed the effects of intensive speech therapy in a nfPPA case. The subject received weekly speech therapy with combined stimulation strategies relating to different components for language processing. Syntactic tasks were applied, comprising construction of sentences based on combinations of worked stimuli and on changing the gender/number/tense of structural elements. The subject showed slight improvements in prosody, fluency and spontaneous speech content, and significant improvements in repetition, reading aloud, and oral-phonatory praxis. This therapy also had a positive impact on other cognitive processes.

A constraint-induced treatment approach implemented with two nfvPPA subjects resulted in improved production of grammatical structures, with maintenance of gains observed at two months post-treatment [31].

Studies on nfvPPA subjects were also directed to speech apraxia [32] and have shown reduction in speech errors through training on text reading.

#### 1.1.3. Interventions Directed to Phonological Deficits

Phonological deterioration starting from a phonological short-term memory deficit characterizes lvPPA. While most individuals with lvPPA mention lexical retrieval problems as their main deficit, some of them are concerned with spelling and short-term memory deficits.

With the premise that the phonological loop is a working memory component, spelling and repetition activities are positive resources used in phonological interventions. Two studies on spelling showed positive results, with learning of phoneme-to-grapheme and phoneme-to-word correspondences [33,34]. In addition, to improve fluency in nfPPA, Louis et al. [35] trained three subjects using a remediation protocol that included auditory exercises that were specifically designed to tackle phonological processing. All participants improved their performance in trained and untrained tasks (generalization to the cookie theft picture and functional communication).

The objective of the present study was to explore intervention techniques for specific language and speech deficits in PPA in a specialized outpatient clinic. Four intervention programs were implemented based on strategies that had shown positive effects in previous studies ([5,7] for reviews), and these were directed to anomia, agrammatism, speech apraxia, and phonological deficits. We investigated the effectiveness of programs in order to generate practice-based evidence and describe the challenges for implementation of these programs in a real-world clinical setting.

## 2. Materials and Methods

### 2.1. Subjects

Over a three-year period, we recruited a convenience sample of individuals with newly diagnosed PPA who were referred to this study by physicians or members of the interdisciplinary team of the Behavioral and Cognitive Neurology Outpatient Clinic of Hospital das Clínicas, Federal University of Minas Gerais, in Belo Horizonte, Brazil. This research was conducted in accordance with the Helsinki Declaration, and participants and their families signed an informed consent statement that was approved by the university’s Ethics Committee.

All subjects included in the study presented diagnosis of probable PPA according to current diagnostic guidelines [2] and had undergone neurological examination and cognitive, speech, and language assessments. These participants were classified into one of three PPA variants (3 nfvPPA, 5 svPPA, and 5 lvPPA), or as mxPPA (5 subjects) presentations. The mean age was 66.3 years, 9 subjects were women, and mean educational length was 14.5 years. The duration of symptoms was 2.1 years.

These individuals with PPA were in mild-to-moderate stages of the syndrome. Their severity of impairment was determined qualitatively. Those who were able to establish functional communication with no need of cues from the therapist were considered to be mild cases. Those who needed support from the therapist, either by simplifying speech to facilitate comprehension or by providing cues to facilitate oral production were considered to be at the moderate stage. Participants with significant functional communication difficulties, such as those unable to give an oral response, or who displayed unintelligible speech were considered to be severe cases.

The inclusion criteria involved a minimum literacy level (at least two years of formal education) and agreement to complete the treatment cycle, be evaluated and undergo post-evaluation. The exclusion criteria involved severe hearing and/or visual deficits, and severe motor or language deficits that would impact the implementation of the programs.

### 2.2. Methods

The subjects were seen at the Behavioral and Cognitive Neurology Outpatient Clinic. The first stage consisted of a medical evaluation (the team included neurologists, geriatricians and psychiatrists), followed by evaluation by a speech therapist and a neuropsychologist. An overall cognitive assessment and a neuropsychological evaluation were used to assist in making the clinical diagnosis and to identify the degree of preservation of non-linguistic cognitive abilities. With these assessments and neuroimaging examinations, the study team assessed the clinical diagnoses and invited participants.

The cognitive and language evaluation for PPA diagnosis and characterization varied among the cases and included some of the tests listed below.

- Overall cognitive assessment and neuropsychological evaluation:
  (1). Mini-mental state examination (MMSE) [36,37];
  (2). Dementia rating scale (DRS) [38,39,40].
- Language assessment:
  (1). Auditory comprehension tests: word and sentence comprehension tests from the Montreal-Toulouse battery (MTL) [41,42] and/or the Boston diagnostic aphasia examination (BDAE) [43,44] and/or the Cambridge semantic memory research battery (CSMRB) [45,46,47] and/or the token test [48,49] and/or the Trog-2 test [50,51,52,53];
  (2). Visual confrontation naming tests: Boston naming test (BDAE) and/or CSMRB;
  (3). Repetition—words, non-words, and phrases of MTL or BDAE;
  (4). Reading words and non-words—HFSP reading aloud test [54];
  (5). Writing words and non-words—HFSP writing to dictation test [54,55,56];
  (6). Reading comprehension: subtests from MTL and BDAE;
  (7). Verbal fluency tasks:
    - Semantic category (animals) [57,58];
    - Phonemic fluency (FAS) [59];
  (8). Recognition and naming of famous faces [60];
  (9). Oral discourse—description of the cookie theft picture [43,61] and correction criteria suggested by Croisile et al. [62];
  (10). Word definition—CSMRB;
  (11). Camels and cactus test of semantic association [63,64];
  (12). Speech praxis protocol [65]; oral agility and oral discourse (BDAE).

Reading, writing, object knowledge and motor speech were assessed qualitatively. For reading and writing assessments, we used the list of words and pseudowords that was developed as part of the HFSP research project. This list was devised in order to study acquired dyslexia and dysgraphia across different written systems and contains words and pseudowords. We analyzed error types in two ways: (a) regularizations (irregular words or commonly used foreign words (e.g., “pizza”) were read or written by applying grapheme-to-phoneme or phoneme-grapheme conversion rules, supported by the auditory representation of the stimuli instead of orthographic memory); (b) phonological or graphemic paralexias and paragraphias (additions, omissions and substitutions, indicative of the dysfunction of grapheme–phoneme conversion mechanisms or working memory deficits).

A different selection of tests for language assessment was applied to each subject. The results from these tests supported classification of the type of aphasia according to the semantic or syntactic losses that were identified, for example. Through this, the most evident difficulties could be identified in order to decide which type of program each individual should be referred to. Some of the tools used for language assessment were not validated for use in Portuguese but were translated, adapted, and applied to a group of cognitively healthy controls. The studies conducted on the versions of the language tests used in the current study are referenced above.

### 2.3. Study Design

This was a multiple single-case study consisting of four stages: (1) complete language assessment and pre-test (trained/untrained items); (2) speech and language intervention (four different types); (3) post-test (trained and untrained items) and, for the naming and sentence production interventions, subjects were also assessed in another task in order to address generalization; (4) follow up, which was conducted 1–8 months after completion of the program.

After the language assessment, each participant was allocated to a cognitive intervention program directed towards a specific language-speech impairment. This program was individualized and was chosen considering: (1) speech and language deficits (the most severe or apparent impairment); and (2) complaints and communication needs, in order to achieve functional adaptation. The functional deficits were identified during the clinical interview with the patients and their families. The best approach was then defined as a consensus with at least two speech therapists.

For the intervention phase, three speech and language therapists adapted four semi-structured programs that was designed for PPA deficits. Rehabilitation materials were personalized, and intervention programs were adjusted to the severity of deficits. The subjects were invited to participate in a 24-session program, but this length of program was not always possible, and the number of sessions was adjusted (see general procedures) to account for any particular mobility issues (for example, whether the individual was living in the city where the clinic was located, could afford transportation to the clinic, or needed a caregiver to accompany him/her, etc.).

The post-test stage consisted of reevaluation on the trained and untrained items (one week after the last rehabilitation session) to assess the effects of the treatment. In addition, for the interventions that focused on naming and on sentence production, generalization was also assessed in a different language task.

The degree of maintenance of the gains (follow up) was assessed in a subgroup of the subjects, at the time when they returned for a clinical consultation. The participants were retested on trained and untrained items. Due to time constraints, in two cases only the trained items were tested.

#### General Procedures

The clinical evaluations and intervention programs were performed by licensed speech and language therapists at the Behavioral and Cognitive Neurology Outpatient Clinic of Hospital das Clínicas, Federal University of Minas Gerais, in Belo Horizonte, Brazil.

The participants were offered 24 sessions of 50 min each, implemented over four months (twice a week). The programs were adjusted according to each individual’s or family’s time constraints in this context of a real clinical setting. In some cases, the subjects were just temporarily visiting the region, to look for a diagnosis in a specialized clinic, and treatment had to be implemented within a period of only two weeks. In other cases, the families committed to a three-month period, while in yet other cases we were able to extend the intervention and see the subjects for 12 months. Because of the heterogeneity of the duration of the treatments provided, as well as the decision to use a tailor-made approach in designing the therapy, we applied a multiple single-case study design in order to report on the effectiveness of the therapies.

Regardless of the type of intervention, the participants were encouraged to practice at home, and we offered training to the caregivers to support this practice (they were trained to assist when the subjects required help), but not all individuals and caregivers were able to follow this procedure. The stimuli sent to their homes were the same as those trained in the sessions. Practice at home was encouraged throughout the treatment; however, it was not formally monitored.

### 2.4. Interventions

#### 2.4.1. Intervention Focusing on Naming

This treatment was based on Senaha et al. [66] and was aimed at naming deficits (either due to semantic memory deterioration or to lexical retrieval deficits). Its main goal was to improve or maintain individuals’ performance in a set of core vocabulary items that could support their communication needs, with a remediation or prophylaxis approach, respectively. The items to be trained were selected for each subject considering: (1) specific needs and relevance to daily life; and (2) relative preservation of semantic knowledge of that item. Items were selected after interviewing the participant, spouse or frequent communication partner before the first week of the study. Before starting the rehabilitation program, the participants’ families were involved in the selection of relevant words for the training. The criterion was their relevance to daily communication. The trained and untrained sets included both correctly named and incorrectly named stimuli that were presented in the pre-test. The only requirement was that the patient was seen to retain some semantic knowledge about the item in the pre-test (i.e., the ability to describe the context within which that item is usually seen, or its function, etc.). The sets included items from different semantic categories. The items consisted predominantly of picturable nouns, proper nouns, adjectives and verbs, as required, depending on the participants’ communication needs. The number of items to be trained varied among the participants and was adjusted to their motivation for intervention (i.e., the amount of time that they could dedicate to daily practices). The training consisted of looking at meaningful pictures or photos of objects or people and trying to name them. The subjects were discouraged from guessing (i.e., following the principles of errorless learning) and were encouraged to check the written corresponding names at the back of each card in case they were not sure. Then, they were asked to read the names aloud and build a meaningful sentence to use that word in context. When the subject was unable to produce this sentence on his own, the therapist elaborated it and asked for repetition. This last step was included in the training routine and differed from the procedure used by Senaha et al. [52]. As the participants’ naming performance improved, the last letters/syllables were gradually erased from the back of the card until only the first letter remained as a written graphic cue that induced correct naming of each stimulus.

After selecting the training set, another set of items was prepared by two or three speech and language therapists for each subject (control set). These included items of the same grammatical category, of similar familiarity and picture complexity as in the training set. The subjects’ performance regarding the trained and untrained items was assessed twice in all cases (one week before and after the intervention). Some participants had a third evaluation (follow-up). The trained items were individualized, but the untrained items were selected from a set of stimuli that the speech and language therapists used for their interventions, which were matched as much as possible to the trained set, according to psycholinguistic parameters (grammatical class, familiarity and visual complexity). The subjects’ comprehension and preservation of some semantic knowledge of the stimuli in the sets was assessed indirectly through qualitative analysis on the responses to naming in the pre-test and the consensual decisions of the speech and language therapists, based on clinical judgment. Retention of basic semantic knowledge of the items in the lists was demonstrated through the ability to provide at least a basic description or show with gestures how to use the item or the context in which it is usually found.

The effectiveness of the intervention was evaluated by comparing the numbers of correct responses before and after the treatment from the trained and untrained (control) items. The generalization was evaluated by comparing the individuals’ performances in another task (semantic verbal fluency), before and after the treatment.

#### 2.4.2. Intervention Focusing on Sentence Production

This intervention was based on Bock and Levelt’s model of sentence production. In our study, the two participants assigned to this treatment received an intervention targeting the positional level. We aimed at verb inflections for production of accurate simple sentences with the structure “subject-verb-object”. We targeted the verb due to its central role in the sentence.

Twenty regular, familiar and high-frequency verbs were selected for the set of training and control stimuli, based on daily routines (examples: to get up, to eat, to cook, to shop, to work, and to go to sleep). We used a set of 40 written sentences with a gap to be filled by a verb in the present or in the past tense (an adverb at the beginning of the sentence cued the verb tense, i.e., “every day” or “yesterday”). Each verb was practiced in both tenses with a model provided by the therapist (repetition). The therapist provided the model aloud (adverb + subject + inflected verb) and the subject was asked to read the sentence and reproduce the verb form in the correct position and inflection (where there was a blank). Then, the therapist asked the subject to produce the full sentence again without reading support. A second drill consisted in providing a written prompt (adverb + subject + verb in the infinitive form) and ask the subject to produce the full sentence. Errors were discouraged; if necessary, the subject could use the written material (i.e., errorless learning). This procedure was repeated until the subject was able to produce the complete sentence accurately from the adverb, subject and verb prompt (e.g., from the prompt “Yesterday + to eat”, the subject should produce “Yesterday I ate a sandwich”). Models and cues were gradually removed until the subjects were able to produce and speak the sentence aloud accurately.

Another 40 sentences with 20 different regular, familiar, and high frequency verbs were used as control set.

The effectiveness of the intervention was evaluated by comparing sentence production before and after the treatment, comparing gains in trained and untrained items. Discourse production from the cookie theft picture was used to look at the transference of the training to discourse.

#### 2.4.3. Intervention Focusing on Speech Production

Based on Henry et al. [32], we implemented a treatment method using structured oral reading as a tool for improving the production of multisyllabic words (two or more syllables). This was directed towards individuals presenting apraxia of speech. During the treatment sessions, the subjects were trained in self-detection and correction of speech errors while reading one text aloud (the training involved rereading of the same text over the sessions). The treatment approach involved the following steps:

- The subject was required to read aloud a selected text. When he/she produced a word incorrectly (with one or more speech sound errors), he/she was asked to stop reading and practice that word (target).
- The subject produced the word syllable-by-syllable many times until he/she reached the correct articulation (appropriate prosody and speed of speech). If the target was a multisyllabic word, it was underlined in the text and lines were drawn dividing the word into constituent syllables. Single-syllable words were repeated until correctly produced in isolation.

After success in producing the word in isolation, the subjects were asked to read the sentence again in order to achieve correct word production in sentence context. If the word was again produced erroneously, the subjects were asked to repeat the previous steps, until the entire sentence was produced correctly.

Two different texts were applied for training (one for each participant), considering that their educational levels were different. The simplest had 120 multisyllabic words and the most complex had 319. The untrained texts had 95 and 179 multisyllabic words, respectively.

For homework, the subjects were encouraged to train on the text used in the session.

The effectiveness of the intervention was evaluated by comparing accuracy in the production of trained and untrained multisyllabic words (pre- and post-intervention). The pre-test intervention measurements considered the number of errors that the subjects made in the first reading of the text. We compared their performance in the trained text with their performance in an untrained text.

#### 2.4.4. Intervention Focusing on Phonological Awareness and Verbal Working Memory

Spelling of words requires temporary storage of the sequence of letters in working memory (graphemic buffer) while the individual letters are being written or spelled out aloud. Moreover, spelling of familiar words in dictation involves recognition of the spoken word (access to the stored phonological representation of the word) and access to the correct spelling of the word (the stored orthographic lexical representation) [56]. Therefore, spelling is used as a strategy for phonological treatment focusing on phonological awareness and verbal working memory, in cases of aphasia [33,67,68].

Phonological deterioration, starting from a phonological short-term memory deficit, characterizes lvPPA. Whereas most individuals with this syndrome mention lexical retrieval problems as their main deficit, some are more concerned with spelling deterioration and short-term memory deficit. Given that there were few studies on lvPPA and, to our knowledge, none reported any treatment addressing phonological deficits and spelling, we developed a protocol based on the study of Louis et al. [35], while also combining some strategies used in individuals with post-stroke aphasia.

The training consisted of activities at the syllable and phonemic levels, along with oral and written spelling. Twenty regular words were selected for the training/control stimuli set. In every session, the subjects practiced the spelling of each word through dictation. If there was an error in the spelling, the therapist guided the subject to read his/her production aloud, so that the subject could try to identify the error and write and/or spell the word aloud again. If the word was misspelled again, visual support was provided (written word) and the subject was asked to copy the word. Other activities were also practiced in the sessions: forming words from a group of syllables or phonemes (synthesis), identifying the number of syllables and phonemes in words (analysis), identifying rhymes and alliterations and manipulating syllables and phonemes to form new words.

Another set of 36 regular words were used as controls.

The effectiveness of the intervention was evaluated by comparing accuracy in spelling pre- and post-intervention. We compared the performance in trained and untrained words.

## 3. Data Analysis and Statistics

The treatment effects were analyzed for each subject using the Wilcoxon signed rank test to determine whether there were any differences in the numbers of correct responses from the trained and untrained stimuli sets from before and to after treatment. We used JAMOVI version 1.6, [69] for the statistical analyses. Since nonparametric tests do not include confidence interval values or effect sizes, we reported estimates generated through paired t tests and Cohen’s d effect sizes in order to estimate the internal validity of the study. However, those measurements should not be considered for generalization purposes. The statistical significance level was set at 0.05 and we reported 95% confidence intervals.

## 4. Results

Thirty-two subjects were referred to the study within the three-year recruitment period. Six subjects with severe language deficits were excluded and eight subjects did not complete the intervention program. In relation to these non-adherent cases, five were svPPA, two nfvPPA, and one mxPPA. The reasons for dropping out from the treatment program included: frustration; anxiety and discouragement due to their own difficulties; illness in the family; unwillingness to do activities at home; distance between the home and the outpatient unit; and feeling that the treatment was not solving the problem.

Eighteen individuals with different PPA variants participated in the study. Table 1 shows the demographic and clinical characterization of the participants and Table 2 shows their performance in formal language tests. For all of them, Portuguese was their first language. None of them had any visual or hearing impairments. All of them were at the mild or moderate stages of the syndrome, and all of them were allocated to one out of the four types of intervention, as mentioned previously. Seven undertook the follow-up assessment. All subjects had at least one cognitive screening and none of them manifested impairment in other major cognitive domains that could significantly interfere with language.

Out of the 18 individuals with PPA who were included in the study, 3 met the criteria for nfvPPA (participants C15, C16, and C17), 5 for lvPPA (participants C6, C7, C8, C11, and C12), 5 for svPPA (participants C1, C2, C3, C4 and C5) and 5 for mxPPA (participants C9, C10, C13, C14 and C18).

Ten participants received an intervention focused on naming (C1, C2, C3, C4, C5, C6, C7, C8, C9 and C10); two received therapy for sentence production (C15 and C16); two for speech production (C17 and C18) and four for phonological awareness and verbal working memory (C11, C12, C13 and C14) (Table 3).

### 4.1. Intervention Focusing on Naming

As shown in Table 3, all the subjects improved significantly with regard to trained items. However, the estimated effect sizes varied from large (C1, C3, and C8) to medium (C2, C5, C9) and small (C4, C6, C7, and C10). The set of trained stimuli varied among the participants: for C3 and C8, an intervention of remediation was implemented in which only items that participants failed to name at the baseline were trained. For the other subjects, prophylaxis items were also included. The number of pictures selected for the training varied among the subjects, depending on the severity of the deficit and the acceptance and motivation to engage in the treatment.

Four participants presented significantly improved performance regarding untrained stimuli: C1 and C5 (svPPA); C8 (lvPPA); and C9 (mxPPA) (Table 4). C1 and C8 received a remediation program in which their pre-test performance was very different between trained and untrained stimuli. The implications of this design for the interpretation of therapy gains are addressed in the discussion.

Generalization to a different task was observed only in C1 and C5 (both svPPA), with marginal significance in C1. Five subjects kept the same level of performance and three declined, but not significantly.

Follow up was conducted in four cases (C1, C7, C9, and C10). Two participants maintained the treatment results and two worsened significantly with regard to trained items. For the untrained items, all subjects maintained the results (Table 5).

### 4.2. Intervention Focusing on Sentence Production

As shown in Table 3, two subjects received this treatment. C15 received prophylaxis treatment and presented no significant change in the trained items (but there was an increase in the correctness of the trained items). There was a significant improvement in the untrained items. This strategy was also implemented in another task, with improvement in the cookie theft picture, but without statistical significance (Table 4).

In contrast, subject C16 received remediation treatment and improved significantly in trained and untrained items with large and medium effects, respectively. However, the strategy was not transferred to discourse, such that there was a significant decline in relation to the cookie theft picture.

A follow-up assessment was undertaken in relation to one participant (C15), one month after the end of the intervention, with maintenance of treatment results, both for trained and for untrained items (Table 5).

### 4.3. Intervention Focusing on Speech Production

As shown in Table 3, both subjects who participated in this intervention improved significantly in relation to the trained and untrained texts. Thus, they presented significant reductions in articulatory errors in multisyllabic words. These participants did not perform tests to assess generalization for other tasks and neither of them returned for the follow-up evaluation.

### 4.4. Intervention Focusing on Phonological Awareness and Verbal Working Memory

The four subjects who took part in this training did not present any significant improvement in spelling after the intervention, either for trained or untrained items. However, correct responses to trained items numerically increased among all the subjects, whereas the number of correct responses to untrained items decreased for three of them and increased for C14.

Follow-up of trained items was possible for C11 and C13. Both participants demonstrated maintenance of the treatment results (Table 5).

## 5. Discussion

This study investigated the implementation and effectiveness of four different interventions for PPA. We used a client-centered approach in which treatments were offered considering the subjects’ main difficulties and concerns, and with individualized relevant stimuli for training. To our knowledge, this is one of the largest case series reporting language intervention results in PPA, and it has strong ecological validity in that it reports on work conducted in a public specialized outpatient clinic. We adjusted the programs to several individual variables involving patients and their caregivers, which is expected to happen in real clinical contexts. Motivation and engagement with treatment were also considered. Thus, some subjects received a more prophylactic form of treatment, whereas others received treatments with more items that involved “relearning” or “reacquiring”.

We acknowledge that the high variability of treatments compromises the generalization and replicability of our results. In addition, as the list of trained and untrained stimuli were not strictly matched according to psycholinguistic parameters or to pre-treatment performance, there are important limitations on interpreting the results from generalization. Therefore, our conclusions and discussions should be considered at the level of “practice-based evidence” [9], in which we observed benefits from SLT in a large sample of PPA subjects. We proposed different interventions addressing not only naming and lexical retrieval, but also other language and speech impairments.

Practice-based evidence can also be demonstrated through case studies of individuals with PPA with gains after intervention [9]. Moreover, the ASHA report of the Joint Coordinating Committee on Evidence-Based Practice [70] argues for the importance of the initial investigation evidence, even when it does not meet rigorous quality standards. That report also mentioned principles of evidence-based practice followed by speech and language therapists that were considered in the present study: client-focused care approach, clear communication to aid the client’s weight clinical alternatives, pursuit of consensus decisions, and top-notch clinical care.

### 5.1. Intervention Focusing on Naming

In our study, all ten subjects (5 svPPA, 3 lvPPA and 2 mxPPA) who underwent this type of intervention improved significantly in relation to the treated items and four also significantly improved in relation to untreated items. Other studies have had similar results and have demonstrated that individuals with PPA are able to relearn target vocabulary during the active phase of treatment [11,12,13,14,15,16,17] and that learning can be generalized to untrained stimuli [13,15,16,18,19,20]. However, the latter result is not consistent across studies. Among our subjects, two svPPA subjects presented generalizations for other language activities (semantic verbal fluency). Our results corroborate the results in the literature [10,71], in that they show that generalization is particularly difficult to achieve in the semantic variant, given that in situations of degraded semantic knowledge, learning is rigid and context dependent. Patients with more evident therapy gains received a remediation program in which pre-test performance was very different for trained and untrained stimuli. In a repeated-measurement design, extreme results tend to regress to the mean. In our study, this statistical phenomenon may have inflated the improvement in treated items, compared with untreated items. Despite this limitation, the gains were clinically significant and confirm the results from previous studies, thus supporting practice-based evidence of a benefit from behavioral interventions addressing naming deficits in PPA.

Four participants underwent a follow-up evaluation, on average six months after the end of the intervention. Two maintained the treatment results and two worsened significantly in relation to trained items. In the untrained items, all subjects maintained their results. Our findings differ partly from those of the systematic review of Cadório et al. [71], which included 25 papers on semantic therapy in different PPA subtypes, encompassing 51 subjects in total. Those authors stated that generalization was more difficult to achieve in the semantic variant (as seen in most of these subjects), compared with the nonfluent and logopenic variants. However, the lack of strict control of psycholinguistic variables, as well as the differences in programs (remediation vs. prophylaxis), limits the interpretation of generalization and maintenance findings from the present study. On the other hand, the personal relevance of the stimuli selected and involvement of the individual with PPA in this selection are factors that may have contributed to the success of the language therapy in the present study, in the same way as in other reports [10,12,14,72].

Similarly to the present study, Croot [8] also studied lexical retrieval treatment among individuals with heterogeneous clinical presentations of PPA. The heterogeneous nature of the sample allowed to observe a range of treatment outcomes and adherence patterns under the same treatment protocol and to describe disease and participant factors associated with these outcomes.

### 5.2. Intervention Focusing on Sentence Production

NfPPA usually presents with mixed symptoms of motor and cognitive-linguistic deficits. Studies on treatments for this variable are less common than on treatments for svPPA. The results show that approaches that focus on the deficit (agrammatism, phonological skills, and speech apraxia, for example) are beneficial to individuals with PPA.

Our two participants who underwent this type of intervention (both nfPPA) improved in relation to both treated and untreated items, thus corroborating previous studies [29,30,31,35,73]. Regarding untrained items, both of them improved significantly.

Only one subject presented generalization for other language activities (cookie theft picture description), with better sentence construction in relation to the pre-test. Schneider et al. [29] and Louis et al. [35] also showed generalization of results for items and untrained material. Cadório et al. [71] show that generalization is easier to achieve in this group of subjects than in relation to the semantic subtype.

One participant underwent a follow-up evaluation one month after the end of the intervention, with maintenance of the results. Among the follow-up studies, only Hameister et al. [31] reported that learning was maintained after the end of therapy.

### 5.3. Intervention Focusing on Speech Production

Few studies have implemented interventions to improve fluency in nfPPA.

Structured oral reading proved to be an efficient and effective means of addressing multisyllabic word production in speech apraxia associated with nfPPA. In the study by Henry et al. [32], one participant showed a reduction in speech errors during the reading of novel text. Similarly, the two subjects in our sample who underwent this intervention (one nfPPA and one mxPPA) improved significantly in relation to both treated and untreated items.

### 5.4. Intervention Focusing on Phonological Awareness and Verbal Working Memory

Among the four subjects (two lvPPA and two mxPPA) treated with this type of intervention, none presented any significant improvement in spelling after the intervention, in relation either to trained or to untrained items. However, all four of them showed numerical increases in the correct responses relating to trained items, whereas three showed decreases relating to untrained items and only subject C14 showed an increase in this regard.

It is noteworthy that maintenance signs of the same level of function in progressive disorders should be seen as a success. Moreover, in these cases it is important to slow down the progression and maintain the communication abilities of subjects [74].

Regarding follow up, two participants were reassessed, with maintenance of treatment results, but without statistical significance. This was comparable with the results of Beeson et al. [75] and Henry et al. [16], but different from Rapp and Glucroft [34], who demonstrated worsened results in the follow-up reassessment.

### 5.5. General Remarks about Treatments and Concluding Comments

We have reported on treatment results for a case series of individuals with PPA. We now discuss some challenges and limitations of our study and other factors of relevance to interventions directed towards speech and language deficits in PPA.

There are few studies on PPA treatment in low and middle-income countries. Like in other studies on this topic, our sample was not large (although larger than in other studies that recruited individuals in the same clinic) and the participants’ characteristics varied considerably even within the same variant of PPA. However, given the relatively low prevalence of PPA, treatment studies on this population usually involve a small number of participants [10].

Another related matter is adherence to treatment. In our sample, eight subjects dropped out of the study before the post-test: five svPPA, two nfvPPA, and one mxPPA. The average number of sessions that they attended was 17.5. Their reasons for dropping out from the treatment comprised frustration, anxiety, and discouragement with their own difficulties, illness in the family, unwillingness to do activities at home, distance between the consultation office and their home and a perception that their speech and language difficulties were not being “solved”.

Jokel et al. [74] stated that many individuals who participate in a group intervention program find it rewarding and positive. Nonetheless, our results show that this finding is not consistent across different samples. Furthermore, there may be a publication bias such that patients who do not adhere to treatments are not included in publications. In our experience, individual treatment was not always motivating and generated frustration and anxiety among some subjects who were aware of their progressing condition, deficits and prognosis from treatment.

Information about participant adherence to treatment requirements is rarely reported in research studies. Taylor-Rubin et al. [76] studied adherence to treatment in the clinical setting in PPA and mentioned that treatment generally requires the person with PPA and their caregiver to play an active role in initiating and continuing the daily home practice. We believe that personalization of therapeutic material and identification with it favors adherence to the rehabilitation program. Thus, the individuals’ involvement in the selection of stimuli may have been a factor contributing to the success of language therapy in the present study and in other reports [12,72]. In our case series, all the stimuli were personalized, with the aim of improving adherence and achieving better functional results. The use of meaningful materials would favor the stronger use of these materials to support functional communication and indirectly increase participation levels. The goal of rehabilitation is to empower people with cognitive impairment and dementia such that they can participate in everyday life in their families and communities in meaningful ways [77].

Taylor-Rubin et al. [76] discussed personal intrinsic factors (such as depression and mood) and treatment-related extrinsic factors (such as time required and duration), along with social factors, which are a combination of intrinsic and extrinsic factors, and how these relate to adherence to treatment. Their results suggested that commencement of treatment while the person with PPA is in the early stages of disease progression may improve adherence and increase the possibility of positive treatment outcomes. However, according to our experience in the public healthcare system, patients take too long to have their first consultation (for reasons discussed below), which limits the chances of always beginning the treatment in the initial stage of the disease.

The initial severity of deficits and the length of time since the onset of symptoms affects the response to treatment, although it is difficult to establish how this occurs. It is coherent to think that the longer the disease duration is, the greater the linguistic impairment will be and hence the greater the treatment limitations. There is considerable inconsistency in reporting the time that has elapsed post-onset and severity levels in the literature on treatments, since the onset of symptoms is not easily defined. Another effort towards treating individuals with PPA consists of interpreting the response to treatment in the context of disease progression, given that a situation of little or no change in the language skills treated may represent a positive outcome, in comparison with the expected decline [21,74].

A significant number of potential participants could not be included in our study because they were severely impaired. One good alternative for these individuals and their families would be orientation and interaction groups, for the exchanging of experiences and counseling, as proposed by Jokel et al. [74] and by Mooney, Beale and Fried-Oken [78]. It is noteworthy that only seven participants returned for the follow-up assessment. In addition to the difficulty in carrying out follow-up treatment studies on individuals with neurodegenerative diseases, due to their cognitive decline [33], the structure of the public care system in Brazil with specialized clinics usually located in large cities, operating within universities, gives rise to further difficulty. Given that several subjects were not residents of the city where the study was conducted and, instead, were there only for diagnosis and intervention therapy cycles, it became more difficult to have them return for follow-up on a regular basis. The initial schedule envisaged carrying out all reassessments three months after the end of treatment. However, this interval varied between one and six months, due to personal, social, and family issues. This factor was referenced in other contexts. Volkmer et al. [28] mentioned barriers to the provision of speech and language therapy services. They argued that many people with PPA are never referred to speech and language therapy services in the first place, due to the lack of evidence that these interventions give clinically meaningful benefit in PPA, and due to the limited specific speech and language therapy services available.

That is also the reason for some of the very short-term cycles of interventions reported in this study. We believe that interventions need to be patient-centered and tailored. Ideally, cognitive rehabilitation programs should be long-term, in line with the progression of the disease and the changing needs of the subjects. However, many individuals do not have access to cognitive intervention clinics or cannot afford treatments. In contrast, some public services need to deal with high demand from patients and cannot provide long-term follow up. For these contexts, brief cycles of intervention and follow up can be an alternative.

We believe that one important contribution of this paper was that it allowed us to share our clinical experience in implementing interventions among PPA subjects. We reported the results from programs and strategies that could be implemented by speech and language therapists as part of a more comprehensive rehabilitation program. Short cycles can be implemented in contexts where patients lack access to full care and to interventions that can be implemented by caregivers. Conversely, in more complete care settings, therapists may combine different strategies according to the needs of the individual.

Other options for interventions with promising preliminary results are being studied. These include neuromodulation, computer-based approaches, the use of social media and electronic devices, and home-based interventions [23,79,80,81]. They may offer more treatment options, even for the most serious cases. For this study, we considered only behavioral approaches that were already reported in the literature, with the aims of increasing the number of published cases and making the level of evidence stronger.

Behavioral interventions in PPA showed improvement of the targeted language function. However, not all of them showed generalizable and long-lasting effects. Tippett et al. [82] pointed out some reasons that would account for these findings: heterogeneity of symptoms and pathological processes, reflected by the different PPA variants, different stages of disease progression at baseline, and variable rates of decline among participants and studies. Moreover, the trained items were individualized in this study, but untrained items were selected from the speech and language therapist’s materials. Thus, the trained and untrained items were not well matched according to the psycholinguistic criteria. Hence, generalization must be considered with caution. It is important to consider the use of more balanced sets (trained and untrained) in future studies. Similarly, the direct treatment gains in the pre- and post-design (for treated items) need to be interpreted with caution for each individual, since we do not know how stable the pre- and post-scores were. Multiple-baseline assessments would provide a better design for the study and must be implemented in future research.

Generalization of treatment gains for untrained tasks may be related to the nature of the intervention and to the use of episodic/autobiographical information [83]. The maintenance of the results achieved in the training does not seem to be influenced by the PPA subtype, but by other factors, such as continuous practice, duration of treatment, and frequency of sessions [71]. All the item sets exhibited a decline in accuracy from the end of treatment to the follow-up evaluation, which was consistent with the degenerative nature of PPA.

Some participants reported having a subjective perception of improvement in functionality regarding communication at the end of treatment. However, as we did not have any means of objective assessment for analyzing this information, we did not include this observation as part of our results. In future studies, we intend to objectively quantify this information.

Another limitation related to the lack of control over practice at home. Differences between participants may have contributed to different treatment results. The absence of supervision of the control stimuli in the patients’ daily life should also be considered as a limitation of the work, since this could potentially interfere with the results.

We recognize that the absence of a control group is a limitation, but we point out that it is a small sample and heterogeneous as to the types of deficits, which makes it difficult to compare patients with and without rehabilitation.

Lastly, we can highlight that this study addressed some important matters: 1. Our study reported on a range of interventions targeted to the individuals’ communication needs; 2. Different treatments were selected for different individuals, determined by the participants’ language symptoms, and not by their PPA variant; 3. Our study had stronger ecological validity because it was implemented in a clinical context and because the number of subjects who did not adhere to therapy and the reasons for this were also reported.

Although PPA is a progressive disorder, both the immediate effects of treatment and, in some cases, the maintenance results, were positive. The results from our study show the effectiveness of specific behavioral interventions even at “low dose” (short-term intervention cycles).

## Figures and Tables

**Table 1:** Sociodemographic and clinical characterization of the patients.

	Sex	Age (Years)	Schooling (Years)	Disease Duration at Treatment Onset (Years)	PPA Variant	Handedness	Brain Atrophy Pattern	MMSE—DRS Mattis
C1	F	60	22	2	Sv	right-handed	Bilateral T	26/30–116/144
C2	M	62	16	2	Sv	right-handed	Bilateral T (more prominent on the left)	24/30–117/144
C3	M	57	21	1.25	Sv	right-handed	Bilateral T	26/30
C4	M	65	20	2	Sv	right-handed	Left FTP	25/30
C5	F	68	16	2	Sv	right-handed	Bilateral T (more prominent on the left)	28/30
C6	F	56	15	1	Lv	left-handed	Bilateral PO	25/30–127/144
C7	M	62	16	2.5	Lv	right-handed	Left posterior P	24/30
C8	F	80	4	2.5	Lv	right-handed	Left posterior TP	10/30
C9	F	67	11	1	Mx	right-handed	Left FTP	27/30–134/144
C10	M	57	11	1	Mx	right-handed	Left FTP	29/30–131/144
C11	F	76	8	1	Lv	right-handed	Left posterior TPO	19/30
C12	F	60	15	4	Lv	right-handed	Right posterior TP	21/30–126/144
C13	M	69	16	2	Mx	right-handed	Bilateral FT (more prominent on the left)	25/30–113/144
C14	F	65	19	3	Mx	right-handed	Bilateral P (more prominent on the left)	29/30
C15	M	66	15	3	Nf	right-handed	Left FT	28/30
C16	M	70	4	1	Nf	right-handed	Left FTP	17/30–115/144
C17	M	75	7	2	Nf	right-handed	Bilateral T	25/30–131/144
C18	F	78	25	4	Mx	left-handed	Volume reduction expected for age	30/30

Note: Legend: F = female; M = male; sv = semantic variant; lv = logopenic variant; nf = nonfluent variant; mx = non-classified/mixed; T = temporal lobe; P = parietal lobe; PO = parietal occipital lobe; TP = temporal parietal lobe; FT = frontal temporal lobe; FTP = frontal temporal parietal lobe; TPO = temporal parietal occipital lobe; MMSE = mini-mental state examination; DRS Mattis = dementia rating scale.

**Table 2:** Performance of the subjects in formal language tests.

	Naming	Verbal Fluency		Repetition Boston Test			Sentence Comprehension		Reading	Writing	Object Knowledge	Motor Aspects of Speech
	Boston Naming (*n* = 60)	Semantic—Animals	Phonemic—F.A.S.	Words (*n* = 10)	Sentences with High-Frequency Words (*n* = 8)	Sentences with Low-Frequency Words (*n* = 8)	TROG (*n* = 80)	Token Test (*n* = 57)				
C1	30	14	27	10	8	8	75	53	Preserved	Preserved	Preserved	Preserved
C2	30	10	26	9	8	8	NA	51	Isolated phonemic paralexias	Graphic paragraphia- substitutions and regularization of foreign words	Preserved	Speech apraxia
C3	10	10	30	10	8	7	NA	NA	Surface dyslexia	Surface dysgraphia	Moderate-severe impairment	Preserved
C4	28	10	13	10	5	3	68	39	Dyslexia with regularization and semantic paralexias	Surface dysgraphia	Mild	Preserved
C5	4	3	29	10	7	8	74	49	Dyslexia with regularization	Surface dysgraphia and phonological paragraphia	Severe	Preserved
C6	41	13	33	10	7	5	68	NA	Isolated phonemic paralexias-inversion	Isolated paragraphia, spelling changes and graphic omission	Severe	Speech apraxia
C7	19	9	9	10	4	4	NA	NA	Phonological dyslexia	Phonological dysgraphia	Preserved	Preserved
C8	15	5	6	10	1	0	NA	NA	Phonological and regularization errors (low education)	Phonological and regularization errors (low education)	Mild impairment	Preserved
C9	36	18	26	10	8	6	67	47	Preserved	Dysgraphia, phonological and graphemic paragraphias, regularizations of foreign words	Mild	Preserved
C10	48	8	16	10	8	7	79	57	Preserved	Regularizations of foreign words	Preserved	Preserved
C11	40	10	13	10	6	5	52	39	Phonemic paralexias-inversion, omission and substitution	Dysgraphia with phonological paragraphia, spelling changes, graphemic and syllabic omission	Mild	Speech apraxia
C12	33	14	21	9	6	6	62	51	Phonological dyslexia	Phonological dysgraphia (phonological paragraphias and graphemic omission)	Preserved	Speech apraxia
C13	24	10	5	8	6	4	NA	30	Morphological and phonological paralexias (mainly) and lexicalization	Graphemic paragraphia-omission and phonological paragraphia	Mild	Preserved
C14	4	13	15	9	4	4	54	41	Phonemic paralexias-omission	Regularization of foreign words graphemic paragraphia- omission and addition and phonological paragraphia	Preserved	Speech apraxia
C15	35	5	2	9	6	4	51	22	Phonemic paralexias-inversion, omission and substitutions and regularization of foreign words	Regularization of foreign words spelling changes	Mild	Speech apraxia
C16	21	3	3	7	0	1	38	12	Phonemic paralexias-omission and substitutions and regularization of foreign words	Graphemic and phonological paragraphias and lexicalization	Severe	Speech apraxia
C17	35	11	16	9	6	1	76	49	Preserved	Regularization of foreign words and spelling changes	Mild	Speech apraxia
C18	40	11	35	10	8	5	76	48	Preserved	Preserved	Preserved	Speech apraxia

Note: Legend: reading and writing (Boston test and HFSP protocol); motor aspects of speech (speech praxis protocol and Boston test); object knowledge (Cambridge semantic memory research battery). NA = not available.

**Table 3:** Results from the intervention programs.

			Trained/Treated Items						Untrained Items					
Type of Treatment	Subjects	Number of Sessions	Number of Items	Baseline Accuracy	Post-Intervention Accuracy	*p*	Confidence Interval—Compared	Effect Size (Estimate) (Cohen’s d)	Number of Items	Baseline Accuracy	Post-Intervention Accuracy	*p*	Confidence Interval—Compared	Effect Size (Estimate) (Cohen’s d)
Naming	C1	20	177	88	166	<0.01	−0.51/−0.366′	0.885	60	30	35	<0.05	−0.155/−0.011′	0.299
	C2	8	60	18	36	<0.01	−0.419/−0.180′	0.649	60	34	32	0.346	−0.0134/0.080′	0.184
	C3	5	43	0	38	<0.01	−0.983/−0.784′	2.725	60	10	6	0.072	0.0016/0.132	0.265
	C4	16	86	63	77	<0.01	−0.2424/−0.083′	0.438	60	28	30	0.346	−0.080/0.013′	0.184
	C5	8	139	89	139	<0.01	−0.44/−0.27′	0.747	60	4	9	0.037	−0.15/−0.011′	0.299
	C6	14	92	37	46	<0.01	−0.160/−0.360′	0.327	60	45	41	<0.01	−0.542/−0.224′	0.625
	C7	12	80	48	64	<0.01	−0.290/−0.110	0.497	20	11	11	NS	NS	NS
	C8	16	30	0	27	<0.01	−1.014/−0.786′	2.95	30	0	21	<0.01	−0.874/−0.526′	1.50
	C9	7	140	108	140	<0.01	−0.29/−0.158′	0.542	60	36	43	<0.01	−0.20/−0.033′	0.360
	C10	11	147	137	147	<0.01	−0.10/−0.02′	0.269	60	48	50	0.34	−0.08/0.0013′	0.184
Phonological awareness and verbal working memory	C11	12	20	6	7	1	−0.154/0.054’	0.224	36	17	14	0.149	−0.011/0.178’	0.297
	C12	19	20	15	16	1	−0.154/0.054′	0.224	36	31	30	1	−0.028/0.084′	0.167
	C13	19	20	6	7	1	−0.155/0.054′	0.224	36	27	18	<0,01	0.101/0.398′	0.569
	C14	23	20	14	16	0.34	−0.244/0.044′	0.325	36	21	23	0.346	−0.134/0.023′	0.239
Sentence production	C15	6	20	17	20	0.149	−0.321/0.0215′	0.409	20	14	20	0.020	−0.520/−0.800′	0.638
	C16	10	20	2	14	<0.01	0.835/−0.364′	1.194	20	1	7	0.020	−0.520/−0.800′	0.638
Speech production	C17	10	120	108	117	<0.01	−0.123/−0.027′	0.284	95	12	47	<0.01	−0.467/−0.269′	0.760
	C18	24	319	277	319	<0.01	−0.169/−0.094′	0.389	179	120	140	<0.01	−0.158/−0.065′	0.354

Note: Legend: NS = not significant.

**Table 4:** Results from the generalization across subjects.

		Generalization to Others Tasks						
Type of Treatment	Subjects	Verbal Fluency—Animals		Discourse Production from the Cookie Theft Picture		*p*	Confidence Interval—Compared	Effect Size (Estimate) (Cohen’s d)
		Pre	Post	Pre	Post			
Naming	C1	14	18	NU	NU	0.072	−0.435/−0.009′	0.519
	C2	14	10	NU	NU	0.073	0.0150/0.0556′	0.609
	C3	12	12	NU	NU	NS	NS	NS
	C4	10	5	NU	NU	0.037	0.0786/0.691′	0.760
	C5	3	9	NU	NU	0.02	−0.775/−0.148′	0.889
	C6	13	14	NU	NU	1	−0.226/0.829′	0.267
	C7	8	9	NU	NU	1	−0.245/0.0907′	0.277
	C8	5	4	NU	NU	1	−0.0907/0.245′	0.277
	C9	18	14	NU	NU	0.072	0.009/0.435′	0.519
	C10	8	8	NU	NU	NS	NS	NS
Sentence production	C15	NU	NU	8	10	0.346	−0.194/0.0343′	0.289
	C16	NU	NU	4	1	0.149	−0.0169/0.257′	0.362

Note: Legend: NU = not undertaken; NS = not significant; Pre = Pre-intervention; Post = Post-intervention.

**Table 5:** Results from the follow-up assessments.

			Follow Up—Trained Items						Follow Up—Untrained Items					
	Subjects	Time Interval (Months)	Number of Items	Post-Intervention Accuracy	Follow-Up Accuracy	*p*	Confidence Interval—Compared	Effect Size (Cohen’s d)	Number of Items	Post-Intervention Accuracy	Follow-Up Accuracy	*p*	Confidence Interval—Compared	Effect Size (Cohen’s d)
Naming	C1	6	177	166	163	0.149	−0.017/0.279	0.131	60	35	35	NS	NS	NS
	C7	8	80	64	48	<0.001	0.263–0.728	0.497	20	11	9	0.163	−0.044/0.244	0.325
	C9	6	140	140	125	<0.001	0.174/0.515	0.345	60	43	42	1	−0.170/0.050	0.129
	C10	6	147	147	144	0.149	−0.018/0.306	0.144	60	50	50	NS	NS	NS
Phonological awareness and verbal working memory	C11	2	20	7	6	0.330	−0.223/0.665	0.224	36	14	NU	NU	NU	NU
	C13	4	20	7	5	0.346	−0.129/0.771	0.325	36	18	NU	NU	NU	NU
Sentence production		1	20	20	20	NS	NS	NS	20	20	20	NS	NS	NS

Note: Legend: NU = not undertaken; NS = not significant.

## Data Availability

The data presented in this study are available on request from the corresponding author.
