# Correction: Machado et al. Cognitive Intervention Strategies Directed to Speech and Language Deficits in Primary Progressive Aphasia: Practice-Based Evidence from 18 Cases. *Brain Sci.* 2021, *11*, 1268

**DOI:** 10.3390/brainsci12101386

**Published:** 2022-10-13

**Authors:** Thais Helena Machado, Maria Teresa Carthery-Goulart, Aline Carvalho Campanha, Paulo Caramelli

**Affiliations:** 1 Programa de Pós-Graduação em Ciências Aplicadas à Saúde do Adulto, Faculdade de Medicina, Universidade Federal de Minas Gerais, Belo Horizonte 30130-100, MG, Brazil; 2 Grupo de Pesquisa em Neurologia Cognitiva e do Comportamento, Departamento de Clínica Médica, Faculdade de Medicina, Universidade Federal de Minas Gerais, Belo Horizonte 30130-100, MG, Brazil; 3 Av Prudente de Morais, 290-Sala 1106, Belo Horizonte 30380-002, MG, Brazil; 4 Grupo de Estudos em Neurociência da Linguagem e Cognição, Núcleo Interdisciplinar de Neurociência Aplicada, Centro de Matemática, Computação e Cognição da Universidade Federal do ABC, São Bernardo do Campo 09210-580, SP, Brazil; 5 Grupo de Neurologia Cognitiva e do Comportamento, Divisão de Clínica Neurológica, Hospital das Clínicas da Faculdade de Medicina da Universidade de São Paulo, São Paulo 05403-000, SP, Brazil; 6 INCT-ECCE (Instituto Nacional de Ciência e Tecnologia sobre Comportamento, Cognição e Ensino), Rodovia Washington Luís, Km 235, São Carlos 13565-905, SP, Brazil

We would like to have these issues corrected to our recent publication [1].

**Text Correction**

In Section 2.1: The published sentence “These participants were classified into one of three PPA variants (3 nfvPPA, 5 svPPA, and 5 lvPPA), or as mxPPA (5 subjects) presentations” should be replaced with “These participants were classified into one of three PPA variants (3 nfvPPA, 5 svPPA, and 5 lvPPA), or as mxPPA (5 subjects) presentations”. We reanalyzed the data of a patient diagnosed with nfvPPA submitted to the sentence production program in a preliminary application of the program [2].

In Section 4.2: The published sentence “C15 received prophylaxis treatment and presented no significant change in the trained items (but there was an increase in the correctness of the trained items)” should be replaced with “C15 received prophylaxis treatment and presented a significant change in the trained items (there was an increase in the number of correct responses in the trained items)”.

**Error in Tables**

Intervention focusing on sentence production: C15 and C16. The information reported in this table referred to the number of correct verbs (n = 20) instead of the number of correct sentences (n = 40). The correct information for these lines of the Table 3 (results of the intervention) are:

**Table 3:** Results from the intervention programs.

			Trained/Treated Items						Untrained Items					
Type of Treatment	Subjects	Number of Sessions	Number of Items	Baseline Accuracy	Post-Intervention Accuracy	*p*	Confidence Interval—Compared	Effect Size (Estimate) (***Cohen’s*** ***d)***	Number of Items	Baseline Accuracy	Post-Intervention Accuracy	*p*	Confidence Interval—Compared	Effect Size (Estimate) (***Cohen’s*** ***d)***
Sentence production	C15	6	40	34	40	<0.05	−0.266/−0.034′	0.415	40	28	40	<0.01	−0.448/−0.152′	0.646
	C16	10	40	4	28	<0.01	−0.759/−0.441′	1.209	40	2	14	<0.01	−0.448/−0.152′	0.646

Intervention focusing on sentence production: C15. The information reported in this table referred to the number of correct verbs (n = 20) instead of the number of correct sentences (n = 40). The correct information for these lines of the Table 5 (Results from the follow-up assessments) are:

**Table 5:** Results from the follow-up assessments.

			Follow Up—Trained Items						Follow Up—Untrained Items					
	Subjects	Time Interval (Months)	Number of Items	Post-Intervention Accuracy	Follow-Up Accuracy	*p*	Confidence Interval -Compared	Effect Size (Cohen’s *d*)	Number of Items	Post-Intervention Accuracy	Follow-Up Accuracy	*p*	Confidence Interval -Compared	Effect Size (Cohen’s *d*)
Sentence production	C15	1	40	40	40	NS	NS	NS	40	40	40	NS	NS	NS

The authors apologize for any inconvenience caused and state that the scientific conclusions are unaffected. The original publication has also been updated.
